# Development of a Mass-Producible Microfluidic Device for Single and Bulk Mycobacteria Investigations

**DOI:** 10.3390/bios15020108

**Published:** 2025-02-13

**Authors:** Adrian J. T. Teo, Jianhui Gu, Alexander Govyadinov, Pavel Kornilovitch, Peiyun Wang, Serene Goh, Nguyen Truong Tung, Zhen Peng, Keith Koh, King Ho Holden Li

**Affiliations:** 1 School of Mechanical and Aerospace Engineering, Nanyang Technological University, Singapore 639798, Singapore; adrian.teojt@ntu.edu.sg (A.J.T.T.); keith.kohlh@ntu.edu.sg (K.K.); 2 HP Singapore Pte Ltd., 1A Depot Close, Singapore 109842, Singapore; jianhui.gu@hp.com (J.G.); peiyun.wang@hp.com (P.W.); wan.ling.serene.goh@hp.com (S.G.); 3 HP Pte Ltd., Corvallis, OR 97330, USA; alexander.govyadinov@hp.com (A.G.); pavel.kornilovich@hp.com (P.K.); 4 Department of Physics, Oregon State University, Corvallis, OR 97331, USA; 5 School of Chemistry, Chemical Engineering and Biotechnology, Nanyang Technological University, Singapore 639798, Singapore; nguy0139@e.ntu.edu.sg; 6 School of Mechanical Engineering, State Key Laboratory of Intelligent Construction and Healthy Operation and Maintenance of Deep Underground Engineering, Sichuan University, Chengdu 610065, China; pengzhen@stu.scu.edu.cn

**Keywords:** microfluidics, bacteria trapping, contactless trapping, *M. smegmatis*, *M. bovis*

## Abstract

We developed a mass-producible microfluidic device capable of long-term observations of single bacilli and bulk bacteria culture interactions for subsequent antimicrobial resistance (AMR) studies. The device provides high consistency across separate devices due to its standardized manufacturing process unlike conventional microfluidic devices. *Mycobacteria bovis* BCG and *M. smegmatis* are trapped within the microfluidic device using minimal equipment and capillary-based techniques, acting as a surrogate model for the highly pathogenic bacteria *M. tuberculosis*. Individual bacilli and bulk bacteria aggregates were observed across a span of ten growth cycles, revealing bacteria growth morphologies alike those in past research. We accordingly propose that this chip would be appropriate for observations of AMR trials involving *M. tuberculosis*.

## 1. Introduction

The Mycobacterium genus, first named in 1896, currently comprises more than 190 named species where some of the most researched species are *Mycobacterium tuberculosis*, *Mycobacterium smegmatis* and *Mycobacterium bovis* BCG [1,2]. In general, mycobacterium are Gram-positive, aerobic bacteria [3] that are rod-shaped, slow-growing [4] and non-motile [5], with the exception of *M. smegmatis* [6]. Research in this genus intensified with the discovery of *M. tuberculosis* in 1882 [2], leading to the revelation of the genus being “among the most important pathogenic bacteria infecting man” [7]. Apart from healthcare research, mycobacteria are a cornerstone of steroid biomanufacturing with *Mycobacterium* sp. [8] and for uses in environmental research on soil health [9].

Typical methods of cultivation require growing on agar plates [9], culture flasks [10] and bioreactors [11]. The investigations herein are subsequently driven towards population-level discoveries like bulk cell–drug interactions and bulk expression of cells across multiple environments [12]. In recent times, however, capabilities in microfabrication have drastically improved, enabling new methods which are able to trap individual bacteria [13]. Single bacterial analyses conducted herein offer certain advantages over traditional bulk analysis by providing new insights in bacterial growth mechanisms and division patterns [14]. Cell-to-cell heterogeneity under different micro-environment conditions can also be observed [15], providing deeper insights into their genome, proteome and metabolome [16]. Such observations are important for antimicrobial resistance (AMR) evaluations as bacterial cell morphology has been previously reported to be influenced by the presence of antibiotics and drugs [17]. Bacterial drug resistance potential have also been correlated to specific gene mutations in drug-resistant cells which also may be observed with morphological changes of certain cells [18]. Thus, the influence of drugs on bacterial morphology remains attractive for researchers. However, it is imperative to first trap and isolate single bacteria before such evaluations can be derived.

Several methods of single bacteria trapping have been previously demonstrated, like using active means like quartz crystals and acoustic waves [19], or passive means like hydrodynamic forces [13]. Microfluidics, being a primary method for bacteria trapping [15], utilizes fluid dynamics in extremely small volumes for biomedical and chemical applications [20]. This method enables the use of less reagent volumes and a lower risk of contamination with the integration of multiple components within a device. These devices can be used to trap cells, bacteria, microparticles and droplets [21], which are subsequently treated with drugs or with culture media, allowing single-cell analysis to be conducted in a controllable environment [13]. These trapping methods have previously been categorized into contactless trapping and contact-based trapping by Deng et al. [15].

In contact-based trapping, bacterial cells come into contact with the surfaces of the trapping regions during the capture process. External energy is not required, as the trapping only relies on hydrodynamic forces and mechanical obstacles to hold the bacterial cells within the aqueous phase. As part of this, capillary-based trapping [22,23] which only relies on capillary forces within small (~2–3 um) microchannels, is used to trap bacteria [15]. Herein, trapping and analysis can be carried out quickly with minimal equipment while also enabling quantification and viability assessment to be performed [24]. Automation can also be employed through using a motorized stage microscope [25]. Cama et al. [23] demonstrated a similar trapping method in 2020 using the “mother machine” as mentioned by Wang et al. [14] in 2010 to trap individual bacilli for growth observations. Although this design is 14 years old, it is still in high demand and commonly used by others. A list of recent devices developed in the past year is provided in Supplementary Materials Table S1.

The devices employed by Wang and Cama are typically fabricated from soft polymers using stereo-photolithography techniques [26], as are most devices used in bacteria trapping. While being a convenient fabrication method, it often leads to variations across multiple chips as fabrication inconsistencies occur. This results in reduced experimental repeatability and reproducibility, with variations in critical parameters, especially highlighted when critical dimensions are in the scale of 1–2 µm. Simultaneously, complications like contamination of components in a device arise during the fabrication process. Therefore, there is a need for highly consistent devices mass-produced within a clean environment. Another device developed by Baron et al. [27] was able to trap bacteria on demand using acoustics; however, it requires expensive equipment setup for the trapping. Finally, both single and bulk analyses of bacteria would provide different insights towards bacteria’s interaction with different drugs, especially for antimicrobial resistance (AMR) evaluation [28]. Thus, the availability of such a device would greatly benefit the industry.

**Figure 1:**
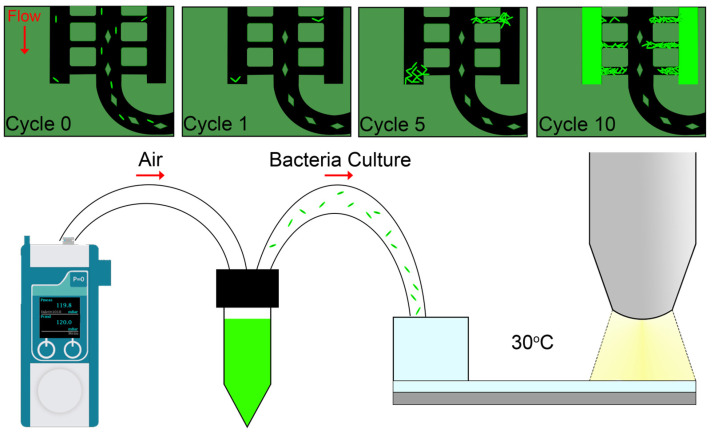
Schematic illustration of experiment setup and observations.

Herein, a microfluidic device developed for single and bulk bacteria growth observations is reported (see Figure 1). This device employs capillary-based trapping through side channels where bacteria are first accumulated upon flowing of a bacteria culture. Subsequent days-long flow of culture media leads to growth of bacteria that creep out of the small channels, which allows for single bacteria observations. The novelty of this device includes the ability to carry out long-term observations on single and bulk bacteria cultures and the capability for mass production. With the introduction of drugs into the microfluidic device, AMR studies can be carried out to provide deeper insights at both the single and bulk bacteria scales. Specifically, target drugs can be flowed into the chip that have bacteria already trapped within. With direct observations of the bacteria aggregates and subsequent quantification of bacteria, this chip would be able to shed light into the effectiveness of said drugs. The bacteria species used in this manuscript are *M. smegmatis* and *M. bovis* BCG, which are the less pathogenic relatives of *M. tuberculosis*. These bacteria models present a suitable surrogate for *M. tuberculosis* infections [29,30]. It is therefore hypothesized that the observations reported here would also be replicable with subsequent use of *M. tuberculosis* bacteria for AMR evaluations.

## 2. Materials and Methods

### 2.1. Device Design

**Figure 2:**
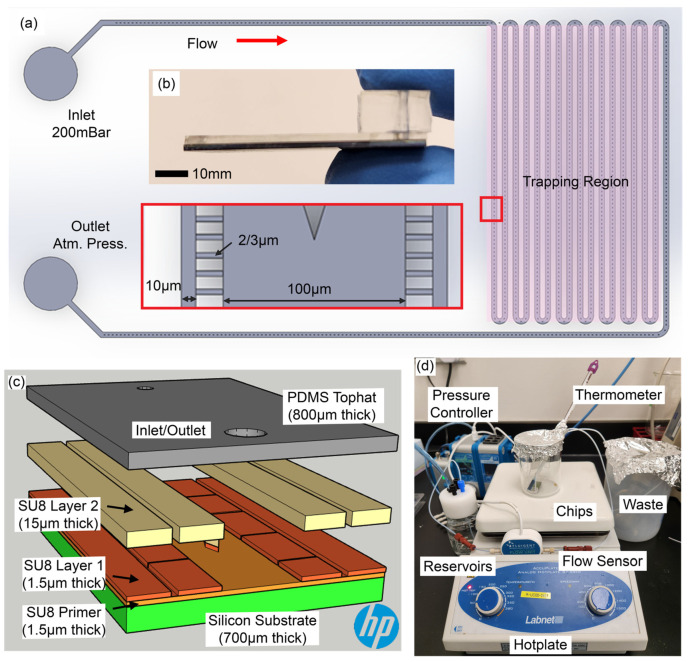
(**a**) Schematic diagram of a microfluidic chip. Insets show the two sections of the device used in flow simulations. (**b**) Image of a final chip used during experiments. The scale bar denotes 10 mm. (**c**) 3D exploded view of layers in chip. (**d**) Experimental setup.

A single device consists of a main serpentine-shaped microfluidic channel that extends from an inlet to a trapping region on other side of the device and then back into an outlet (see Figure 2a). The unique design of the chip was chosen to provide enough space for the positioning of the microscope objective above the trapping region for observation purposes. Within the trapping region, the main microfluidic channel consists of bends and straight paths where the latter includes approximately thirty-nine hundred microchannels each for trapping of individual bacteria. These microchannels are then linked to a side channel where bulk trapping of bacteria can be carried out. By design, the microchannels along the first three bends are 3 μm wide, whereas the subsequent three bends have 2 µm-width branched microchannels for design optimization purposes. The final dimensions of the trapping region were determined based on the consideration of maximizing the number of devices fabricated within a single 8″ wafer. The heights for the main channel, microchannel and side channel are 16.5 µm, 1.5 µm and 16.5 µm, respectively. The height of the main channel was purposely set to be much higher than the height of the microchannels to ensure that bulk flow of the fluids would be directed along the main flow path and not into the microchannels, which would increase the chances of clogging upon bacteria agglomeration. The height of the microchannel was to prevent overlapping of bacteria which would affect observation. The height of the side channel was to be similar as the main channel to reduce fabrication steps. The width of each of those channels are, respectively, 100 µm, 3 or 2 µm, and 10 µm. The critical microchannel dimensions (3 and 2 µm) were chosen to enable single bacilli entrapment within each microchannel. These dimensions are chosen based on the mother machine design mentioned by Wang et al. [14] and fabrication limitations. Diamond structures are also designed in the main microfluidic channel to provide support for the laying of a top hat as the width of the main channel was wide. This top hat effectively converts the exposed microfluidic channels into a completely sealed device for use. Flow simulations also indicate negligible influence of the pillars during bacteria culture or media culture flow (see Supplementary Materials Figure S1).

### 2.2. Chip Fabrication

Microfluidic chips (see Figure 2b) were fabricated in two stages. The first step involved fabrication of microfluidic channels on a silicon wafer substrate. Using photolithography techniques, the microchannel designs are produced using a series of SU8 films of different thicknesses. A primer layer was first coated on an 8″ wafer to improve adhesion of subsequent layers. A subsequent layer is coated for the small branches that connect the main microfluidic channel to the side channels with a thickness of 1.5 µm (see SU8 Layer 1 for Figure 2c). A final 15 µm SU8 layer is subsequently set above the developed designs to extend the height of both the side and main microfluidic channels (see SU8 Layer 2 for Figure 2c). General procedures for SU8 film deposition and patterning can be found elsewhere [31,32]. In total, approximately 70 devices can be fabricated on an 8″ wafer. The wafer is subsequently diced before the second fabrication stage.

The second fabrication steps were to seal the microfluidic device with the adhesion of a final top layer. Several materials were tested (see Supplementary Materials), leading to the selection of polydimethylsiloxane (Dow Corning, Sylgard 184 Silicone Elastomer Kit, USA) membrane. The ratio of crosslinker to elastomer was 1:10. The mixture was spin coated (Laurell Technologies Corporation, WS-650MZ-23NPP, USA) at 500 rpm for 30 s on a petri dish to produce a thickness of 120 um. It was left to cure in a 70 °C incubator (HCS Scientific & Chemical Pte Ltd., Innucell, Singapore). Strips of the membrane were then cut out for bonding to the 53 mm × 14 mm microfluidic chip. Air plasma bonding (Harrick Plasma, PDC-002, Singapore) was implemented at 30 W power for 2 min before being treated with 3-Aminopropyltriethoxysilane (Sigma Aldrich, APTES, Singapore) for 4 min. After cleaning the membrane with warm ethanol, the membrane was manually aligned onto the SU8 layer mentioned earlier, creating the PDMS top hat over the microfluidic channels as shown in Figure 2c. As no critical features were crafted on the PDMS membrane, no alignment issues arose during the process. Finally, a separate 70 µm thick PDMS block was air plasma bonded over the PDMS top hat for temporary connection of tubing.

### 2.3. Growth Conditions and Culture Preparation

Green florescence expressing recombinant strains of *M. smegmatis* mc(2)155 harboring pJKD2893 plasmid and *M. bovis* BCG Pasteur harboring pEGFP plasmid were grown at 37 °C using Middlebrook 7H9 media (Sigma Aldrich, Singapore) supplemented with Album–Dextrose–Saline (ADS). The excitation and emission peaks for the mGreenLantern protein used here are 503/514 nm. Kanamycin was added at a concentration of 50 µg/mL and Gentamicin at 10 µg/mL as selectable marker for pJKD2893 and pEGFP, respectively. Exponentially growing culture (OD600 = 0.2–0.6) were centrifuged at 3800 rpm for 10 min and resuspended in fresh media, followed by additional centrifugation step at 100 rcf to remove multicellular aggregates. Opaque supernatant was carefully decanted and resuspended to a final cell density of ≈10^6^ cells/mL. This cell suspension was transferred to a reservoir and used remain capped throughout the experiment.

### 2.4. Experiment Setup

The experimental set up is depicted in Figure 2d. A pressure controller (Fluigent, Flow EZ, Singapore) is used to provide pressure for driving fluids from the respective reservoirs into the microfluidic device. The pressure controller is first attached to a reservoir filled with 20 mL of culture media and maintained at 200 mBar for 1 h to prime the microfluidic device before introducing the bacteria culture. To fill the bacteria culture, 1 mL reservoirs filled with the bacteria culture are replaced with the media reservoir and flowed at a pressure of 50 mBar for 3 h. The microfluidic chip is connected to a waste reservoir throughout the experiment. A flow sensor (Fluigent, Flow Unit M) is integrated in the inlet tubing before the chip for flow rate measurement. It was experimentally measured that pressurizing at 200 mBar delivers a flow rate of 0.12 µL/min or 5.5 × 10^−3^ m^3^/s within the device. This result was subsequently used for simulations.

Throughout the experiment, the bacteria chip remained housed in a glass beaker on a hot plate (Labnet International, Accuplate, USA) maintaining an ambient temperature of approximately 30 °C. The experiments with *M. smegmatis* were performed with a Zeiss Axios Zoom (Zeiss, USA) microscope with up to 100× magnification, while the experiments with *M. bovis* BCG were carried out with a Leica DM LFS with a series of objectives of up to 63× magnification. The duration of each experiment was dependent on the doubling times of each bacteria. Using *M. bovis* BCG, as the doubling time was approximately 23 h [33]; the duration of each experiment was approximately 5 days for a total of 5 growth cycles. Using *M. smegmatis*, as the doubling time is approximately 3 h [34], the duration of each experiment was approximately 30 h with a total of 10 growth cycles. Microscope images were captured for each experiment with the bacteria chip transferred unto the microscope and captured with 10× objective lens.

## 3. Results and Discussion

**Figure 3:**
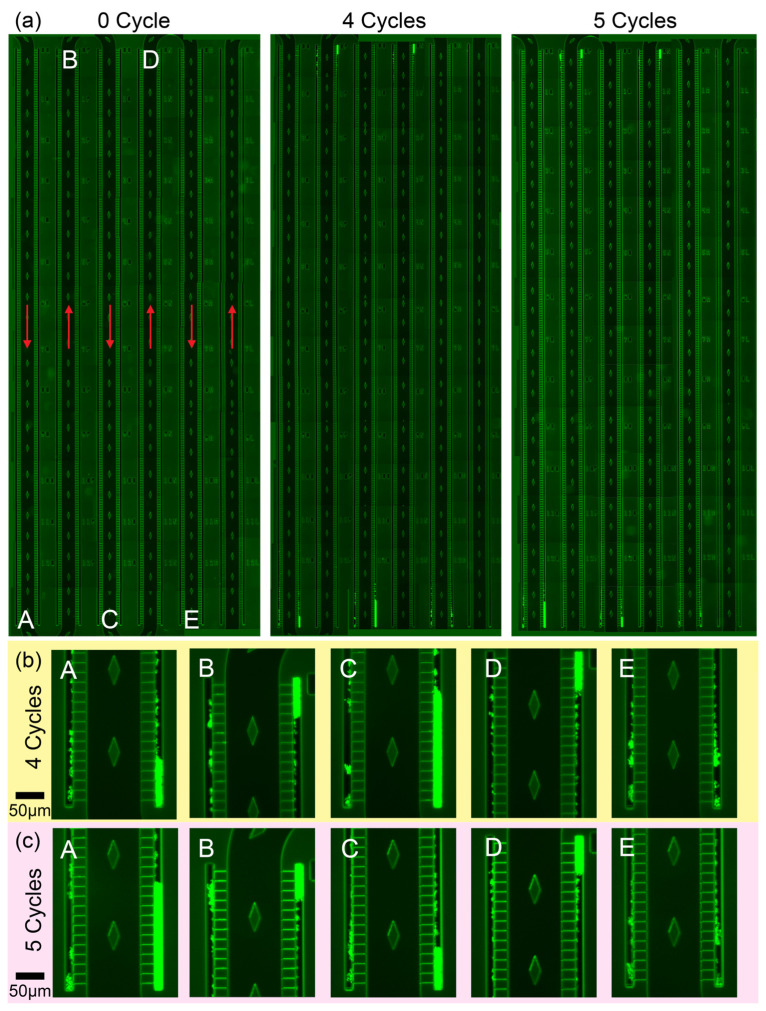
Fluorescence images of a device showing regions of bacteria concentration on the microfluidic chip using *M. bovis* from Day 0, Day 4, and Day 5. (**a**) Magnification images (10×) of locations A, B, C, D, E are given here with flow directions of each channel given in red. (**b**) Magnification images (40×) of trapped clusters after 4 Cycles. (**c**) Magnification images (40×) of trapped clusters after 5 Cycles. With the exception of D, all the locations demonstrate a slight increase from Day 4 to Day 5. Location C increased greatly during this period, suggesting accumulation of bacteria from earlier channels. Location D decreased greatly, suggesting outflow of bacteria from the cluster. The scale bar size is 50 µm.

Individual microscope images were stitched together manually across the trapping region of the microfluidic device to reveal trapping patterns within a single device (see Figure 3a). At the start of the experiment (see 0 cycle in Figure 3a), bacteria were unable to be identified at this magnification as these were trapped individually within the branch microchannels. Separate experiments were carried out below to reveal trapped bacteria at a slightly higher magnification below. After leaving the device to run, observations at the end of the 4th cycle revealed that the majority of the trapped bacteria were found at the side channels before the entrance of each bend (see Figure 3b). This trapping phenomenon was in accordance with observations from simulations as given in Supplementary Materials Figure S2.

In the simulations, particles were simulated to flow within the main microfluidic channel at both the straight and bends of the channel. The particles used here were spherical with a concentration that was similar to the bacteria concentration mentioned above. During the simulation for the straight channel, particles were observed to flow into the side channels via the branch microchannels effortlessly where trapping will occur due to the adherent nature of bacteria. However, after an extended duration of time, the particles were also observed to exit through the same branch microchannels due to the flow dynamics of the system. A simulation video is provided in the Supplementary Materials (see “Video S1 Particle_Motion_Video.mp4”). This phenomenon would suggest that there is no trapping of bacteria. However, the experimental results suggest otherwise, which we attribute to the limitations of being unable to simulate the strong adherent forces produced by bacteria on surfaces. With the inclusion of this force, it is theorized that the bacteria would induce an additional inertia to flow that causes the bacteria to leave the flow streamline and accordingly become trapped in the ends of the side channels. This phenomenon is also the same for the simulations at the bends of the device where the bends do not induce additional effects on the trapping.

**Figure 4:**
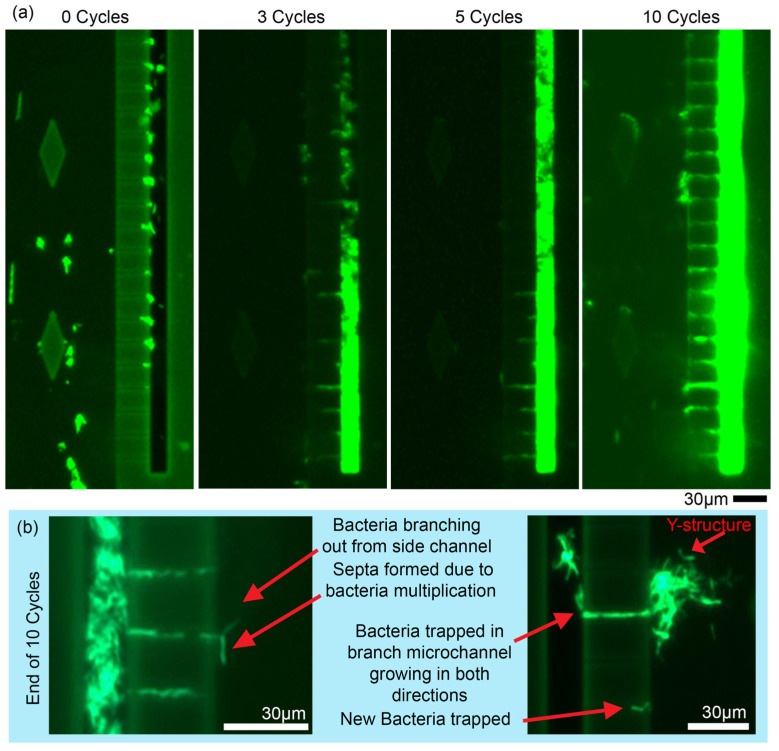
(**a**) Observation of trapping and growth of *M. smegmatis* across 10 cycles. Trappings observed at the edges of individual microchannels at 0 cycles, which formed clusters and started to fill up entire side channel. RLU measurements gave results of 0.84, 3.49, 6.61, and 15.59, respectively. (**b**) Observations from separate experiment with single bacilli growing out of microchannels with new bacilli trapped in microchannel. Overgrowth of bacteria also observed, forming a cluster at microchannels in the right image. The size of the scale bars is 30 µm.

Further growth of these trapped bacteria was evident after 5 growth cycles (see Figure 3b). As the bacterial suspension was moving in from the inlet at the top left corner of the chip, most of the bacteria were trapped in the first few channels, leaving less bacteria available for the later parts of the trapping region. This was observed from the decreased lengths of trapped bacteria aggregates across locations A, B, C, D, E within the same timeframe in Figure 3b. However, during the flowing of the culture medium after the bacteria culture, it was also observed over the duration of the experiment that new bacteria aggregates were formed in previously uninhabited branch microchannels. This additional phenomenon was understood to be due to bacteria originating from earlier clusters that broke off due to the constant flow. These bacteria accordingly travelled into these uninhabited branch microchannels and subsequently got trapped there. These new bacteria may also repeat this process of forming clusters, breaking off, and flowing into subsequent sections of the trapping region by flowing out of the side channels as simulated (see Figure 4b).

Experimental observations of *M. smegmatis* were performed as a representative of mycobacteria with fast doubling rates with a total of 10 growth cycles. A close look at the channels reveal trapping of bacteria at the side channels for *M. smegmatis* across ten growth cycles (see Figure 4a). At 0 cycles, attachment of the bacteria to the branch microchannels was more obvious unlike the experiments with *M. bovis*. The bacteria here was also observed to be in clusters, rather than single bacilli. However, after three cycles, the bacteria were observed to fill up the bottom of the side channel, up to 200 µm length of the side channel. After 5 cycles, the bacteria aggregate to twice the length of the microchannel. After 10 cycles, the bacteria colony in the side channels grew much brighter with the bacteria culture growing out of the microchannel branches. The intensity of the mGreenLantern fluorescence increased gradually, indicating the *M. smegmatis* was growing exponentially within the trapping chip. Relative Light Unit (RLU) measurements at these regions indicate the growth of bacteria with normalized values increasing from 0.84, 3.49, 6.61, and 15.55 from 0 cycles to 10 cycles. Images from a repeat experiment are given in Figure 4b where bacteria grew out of the side channels as individual bacilli. A separate aggregate is also observed as given in Figure 4b to show the growth and extension of the bacteria aggregate out of the branch microchannel. The left image showed an extended bacilli with a middle portion that is slightly less illuminated, indicating a septa developed during multiplication similar to SEM imaging observations by Zaragoza-Contreras et al. [35]. A similar y-shaped structure after bacterial multiplication is observed in the right image indicating multigenerational asymmetric growth of *M. smegmatis* [36]. Critical positioning information and cell orientation information can be extrapolated from these images. With higher resolution imaging, cell dimensions before and after division can accordingly be determined. Subsequent introduction of drugs and antibiotics on such aggregates would thus maximize the exposure of the bacteria to the drugs that are flowed in, effectively revealing changes in bacterial morphology, which can be accordingly observed here.

Throughout experimentation with *M. smegmatis*, biofilm was readily formed in the main microchannels even when flowed at higher pressures of 300 mBar. This biofilm was observed to form as the bacteria stuck to the surface of the SU8 walls amidst high shear stresses as pointed out by Tsagkari et al. [37]. Notably, this interesting phenomenon was not observed in *M. bovis* BCG. We, however, are able to conclude that this device may also be useful for investigating such biofilm formations [38].

**Figure 5:**
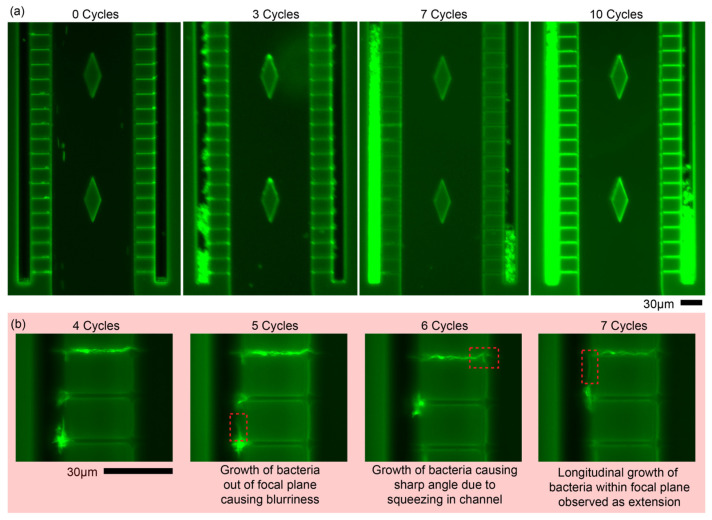
(**a**) Observations of trapping and growth of *M. bovis* BCG versus multiplication cycle. Similar to *M. smegmatis*, weak trapping was observed at 0 Cycles, which gradually increased due to growth. Accumulation of bacteria observed after 3 Cycles where bacteria are trapped at bottom of side channels. Bacteria continued to grow where it subsequently filled the side channels, as observed after 7 Cycles. RLU measurements were observed to gradually increase from 0.35 to 2.17, 10.09 and 12.33 after 10 cycles. (**b**) Individual observations of small clusters allow observations of individual bacilli growing out of individual clusters as given by the red rectangles. The size of scale bars is 30 µm.

Similar and growth observations with *M. bovis* BCG are given in Figure 5. Observations at 0 cycles revealed small amounts of trapping with most of the bacteria flowing downwards. In these channels, small bacteria clusters were observed to be emerging in the left side channel after 3 cycles with huge growth from the 3rd cycle to the 7th cycle (see Figure 5a). Additionally, in the right microchannel, growth was also observed after the 3rd cycle, and subsequently growing as observed up to 10 cycles. Experiments were discontinued thereafter as the bacteria gradually started to fade out, suggesting death. The RLU measurement revealed gradual increments from 0.35 to 2.17, 10.09 and finally 12.33 for the given regions showing growth of the bacteria within the device. Observations from a repeat experiment is given in Figure 5b showing single bacilli growth within the chip. With careful monitoring, each microchannel is able to provide such observations, indicating its usefulness as a platform of assessing drug treatments at the single bacilli level and also at the bacterial population level. Considering each side channel in Figure 5a as an individual bacterial colony, it is hypothesized that the effects of subsequent drug interactions can be observed with the reduction of the luminance progressing from the boundaries of the colony. The speed and efficacy of drugs used can also be determined through this means.

## 4. Conclusions

In summary, the device demonstrated in this manuscript has been demonstrated to be mass producible with high consistency (approximately ~70 devices within an 8″ silicon wafer) and high trapping efficiency where both single bacilli and bulk bacteria aggregates can be observed. Long term observations of the growth of different bacteria can be observed where in this manuscript both *M. smegmatis* and *M. bovis* BCG were observed with durations observed across ten growth cycles for the former and five growth cycles for the latter. These observations have proved to show the growth of individual bacteria and clusters across these time spans proving to be highly suitable for separate studies like antimicrobial studies and drug therapies on bacteria or biofilm formation [38] that are time dependent. A slight disadvantage is in the autofluorescence of the SU8 material and lowered resolution of the images which can be circumvented with current imaging technologies that are beyond the scope of this manuscript.

The technique described here also considerable reduces the need for additional equipment and reagents (function generators [39], acoustic amplifiers [40], optical tweezers [41], and magnetic [42] or gold particles [43,44]) that are not conventionally found in biological laboratories as compared with most recent publications. This promotes its uptake as a convenient method of bacterial observation, especially in higher security Biosafety Level 3 laboratories, especially for studies involving highly pathogenic bacteria. Finally, this device is also capable of demonstrating the same passive bacteria concentration implemented by Han et al. [45] for single-point confocal Raman spectroscopy too. In their discovery, a similar nanogap trap was developed using silicon and glass substrate to trap bacteria for subsequent label-free identification. By using the device described here, multiplexed results can be achieved where multiple clusters could then be identified, increasing the accuracy of data captured. Future works for this device include the utilization of the device with drugs like bedequiline and telobac and finally the trapping of *M. tuberculosis* within for further drug evaluations.

A great need for investigating antimicrobial resistance in bacteria further drives the urgency for developing mass producible devices that enable long-term observations of bacteria, both at a singular and bulk cluster level. The mass production of such devices facilitates more reliable quantitative data collection, reducing the likelihood of errors caused by variability. The device demonstrated here was tested with two bacteria strains, *M. smegmatis* and *M. bovis* BCG. These two strains were specifically chosen due to physical similarities with the highly pathogenic mycobacteria *M. tuberculosis*. The trapping within the device allows observations of single bacteria morphology and growth patterns upon addition of new drugs. Bacteria clusters can also be formed to allow larger scale observations of drug resistance studies. We conclude that this device will benefit future drug discovery works with high consistency and reliability across multiple experiments. The device will also prove to be useful for antimicrobial resistance studies of target mycobacteria.

## Data Availability

All data are provided in the manuscript.

## Supplementary Materials

The following supporting information can be downloaded at: https://www.mdpi.com/article/10.3390/bios15020108/s1, Table S1: Comparison table of recent devices developed; Figure S1: Simulation results (a) with diamond pillar and (b) without pillar; Figure S2: Screenshot of simulation video showing motion of particles within; Video S1 Particle_Flow_Video.mp4. References [46,47,48,49,50,51,52,53,54,55,56,57,58,59,60,61,62,63,64,65,66,67,68,69] are cited in the supplementary materials.
