# Supplementary material for: Development of a Mass-Producible Microfluidic Device for Single and Bulk Mycobacteria Investigations
Source: Biosensors (Basel). 2025 Feb 13;15(2):108. doi: 10.3390/bios15020108 (PMC11853077; doi:10.3390/bios15020108)
Supplement: Supplementary file 1 [file biosensors-15-00108-s001.zip › Supplementary Material for Manuscript.pdf]

Supplementary Material for Manuscript

1. Table for comparison with recent state-of-the-art microfluidic bacteria trapping devices

A recent search on Scopus with the keywords “Bacteria” and “trapping” and “Microfluidics” returned a total of 86 articles whereby 27 publications are based on contact-based methods of trapping. These publications are listed here below with the described device for comparison purposes.

Table S1. Comparison table of recent devices

Authors	Year	Title	Approach	Substrate	Bacteria used	Application
Tanaka D.	‘23	[1]	Branch microchannels	PDMS on glass	Salmonella enterica serovar Typhimurium and Staphylococcus aureus	Bacteria Culture
Khaji Z.	‘22	[2]	Branch microchannels	PDMS on glass	Escherichia coli	Development of integrated cooling system
Forsyth B.	‘21	[3]	Branch microchannels	PDMS on glass	Escherichia coli, Klebsiella pneumoniae, Enterococcus faecalis, and Staphylococcus aureus	Antibiotic susceptibility test
Cama J.	‘20	[4]	Branch microchannels	PDMS on glass	Escherichia coli	Antibiotic susceptibility test
Long Z.	‘13	[5]	Branch microchannels	PDMS on glass	Escherichia coli	Development of microchemostat for high-throughput intracellular measurements.
Wang P.	‘10	[6]	Branch microchannels	PDMS on glass	Escherichia coli	Bacteria behavior studies
Asayes h F.	‘17	[7]	Bubble interface	PDMS on glass	Pseudomonas fluorescens CT07	biofilm studies
Zheng H.	‘23	[8]	Buoyant forces	PDMS on glass	Escherichia coli	Bacteria behavior studies
Spagnol ie S.E.	‘15	[9]	Colloid trap	Theoretical modeling	-	Bacteria behavior studies
Savoran a G.	‘22	[10]	Fluid viscosity	PDMS on glass	Pseudomonas aeruginosa	Bacteria behavior studies
Chang K.-W.	‘19	[11]	Membrane	PMMA with PC membrane	Escherichia coli	Antibiotic susceptibility test
Krafft B.	‘21	[12]	Membrane filter	PDMS on glass with PCTE membrane	Escherichia coli DH5α and Pseudomonas taiwanensis VLB120	disposable optical platform for reliable and rapid pathogen detection
Nam Y.-H.	‘19	[13]	Membrane with nano-slits	PDMS on glass with PDMS membrane	Escherichia coli	Size based trapping
Kim M.-C.	‘10	[14]	Micropillars	Simulations	Escherichia coli	Bacteria behavior studies
Liao C.-C.	‘21	[15]	Microwells	PDMS on glass	Escherichia coli	Early antibiotic therapy for fighting bacteremia
Tokárov á V.	‘21	[16]	Movement across various angled microchannels	PDMS on glass	Vibrio natriegens, Magnetococcus marinus, Pseudomonas putida, Vibrio fischeri, and Escherichia coli	single-cell genomic screening, bacterial entrapment for diagnostics, or biocomputation
Vasdeki s A.E.	‘13	[17]	Single microchannel	PDMS on glass	Escherichia coli	Single cell analysis
Molaei M.	‘16	[18]	Surface shear force	PDMS on glass	Escherichia coli	Bacteria behavior/ biofilm studies
Ganzing er K.A.	‘20	[19]	Trapping chamber	PDMS on glass	-	Production of deformed Giant Unilamellar Vesicles
Gruenb erger A.	‘13	[20]	Trapping chamber	PDMS on glass	Corynebacterium glutamicum	Development of platform for time dependent phenomena of single bacteria

Galon C.M.V.	‘22	[21]	Trapping structures	Simulations	Escherichia coli and Candida auris	Biomedical assays modeling
Probst C.	‘13	[22]	Trapping structures	PDMS on glass	Escherichia coli	Bacteria behavior studies
Chatzi michail S.	‘24	[23]	Tunable backpressure	PDMS on glass	Escherichia coli and Staphylococcus aureus	antimicrobial susceptibility testing
Zhang B.	‘23	[24]	Vertical grooves	PDMS on glass	microparticles	Bacteria behavior studies
Zhang B.	‘22	[25]	Viscoelectic creep	Hydraulic filtration system	Giardia	Parasite behavior studies
Wen K.	‘24	[26]	Vortex	PDMS on glass	Escherichia coli	biofilm studies
Tanasij ević I.	‘22	[27]	Vortex	Simulations	-	Bacteria behavior studies
Ours			Branch microchannels	SU8 on silicon wafer with PDMS cover	Mycobacteria smegmatis and mycobactyeria bovis BCG	Development of device for antimicrobial resistance testing

The main advantage our device pose is in the ability to carry out mass fabrication whereas other devices utilizing the same approach are all using PDMS on glass substrate. This is well known to cause issues like discrepancies across different devices and also pressurization of channels due to its flexibility. By limiting the use of PDMS in our device, it enables less variation of pressure induced while also enabling standardization and reliability of experimental protocols.

2. Material Choice for Top hat

A list of choice materials were identified via literature review for the sealing of the microfluidic device, namely glass, SU8, silicone adhesive films and finally PDMS. Initially, a thin layer of SU8 was used to lay over the microfluidic channels at HP Inc, as part of the chip fabrication process. This method resulted in the need for additional vent holes in the main microfluidic channel for the removal of sacrificial material during the fabrication process. This accordingly led to more issues in the experiment whereby the fluids leaked out through the vent holes in the middle of experiments. Glass coverslips were tested and cut using a laser cutting machine, however bonding proved to be difficult as previously documented methods required the use of high pressure and temperature, which effectively destroyed the prefabricated SU8 microchannels sandwiched between. The use of adhesive films here also resulted in random trapped bubbles and occasional sealing of main microfluidic channels due to excessive pressing. As such, PDMS was the most ideal material as it provided a strong bonding with SU8 upon APTES treatment. However, as PDMS was soft and flaccid at thin layers, diamond pillars were constructed within the main microfluidic channels to provide support (See Figure S1).

3. Flow simulation results for effects of diamond pillars

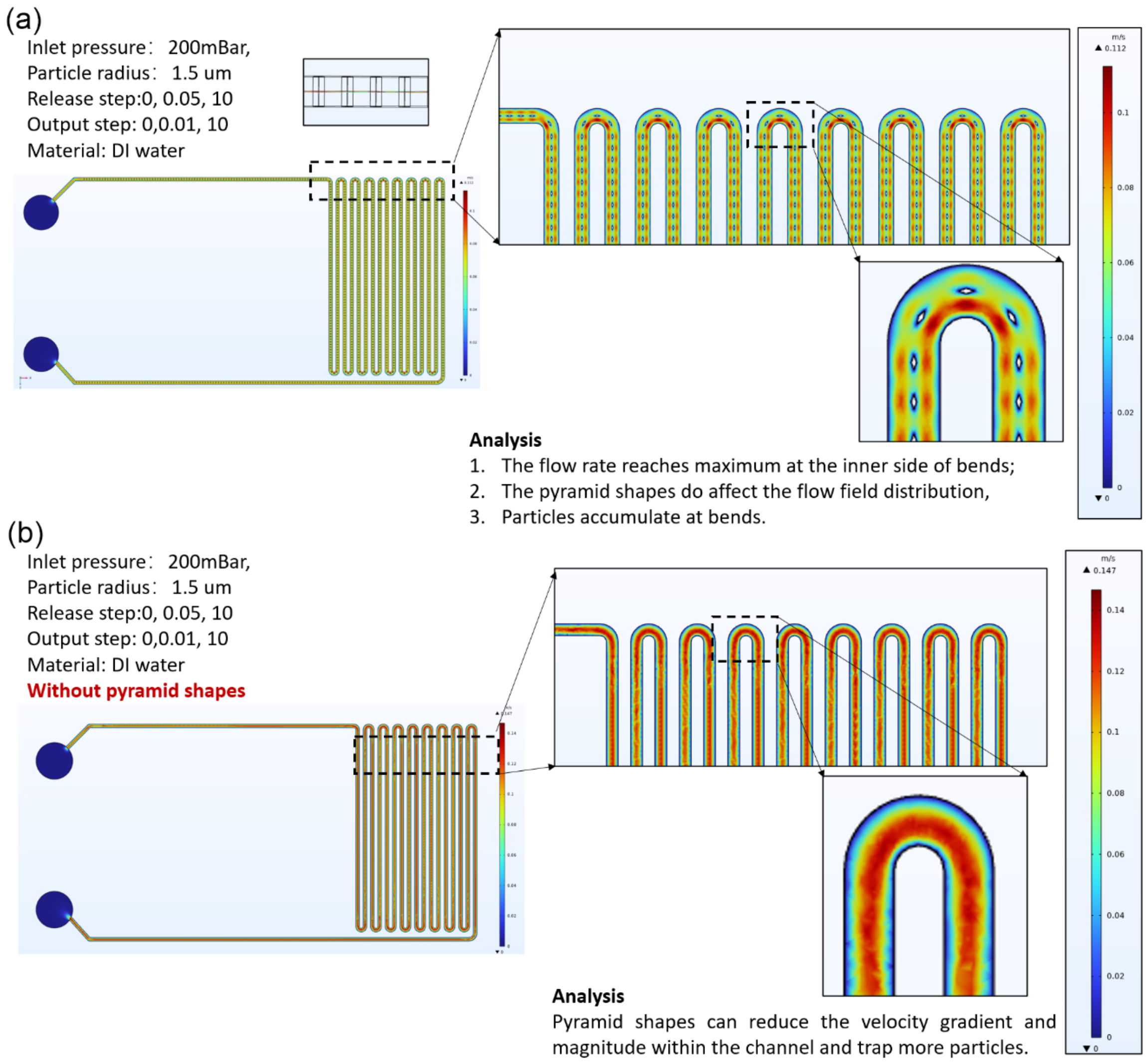

Figure S1: Simulation results of (a) with diamond pillar and (b) without pillar

Simulations with and without the diamond pillars did not reveal much drastic changes in the fluid flow and particle motions. Based on the simulation results, there was an approximately 23% decrease in the average fluid speeds due to the diamond pillars. The average speed with pillars was approximately 0.056m/s as compared to 0.074m/s without pillars. With lower flow speeds, it is hypothesized that the bacteria would be more able to flow into the perpendicular microchannels along the sides thus promoting trapping of bacteria. Flow field observations also reveal that the medial region of highest speed was split symmetrically around the pillars. The diamond pillar designs are also observed to not induce vortices within the microchannels, thus being the best option.

4. Flow simulation video for straight channels

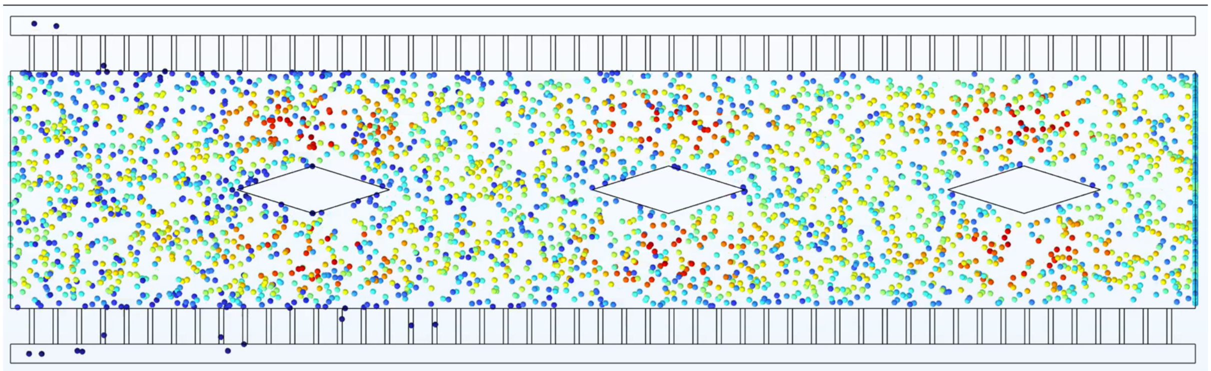

Figure S2: Screenshot of simulation video showing motion of particles within

Simulation video (Video S1 Particle_Flow_Motion.mp4) reveal that particles are easily filtered in and out of the channels. The settings for the simulation are based on assumptions like laminar flow with no-slip boundary conditions, deionized water properties for the fluid, environmental conditions at standard room temperature and pressure, assumption of non-newtonian and non-compressible fluid with pressure driven flow. For the particles, an assumption of 1.5um diameter spherical particles are assumed. The shape of the particle remains a major limitation of the simulation software. As the bacteria observed here are not spherical but rod-shape, there may be small influences in the trapping phenomenon, however the general flow would remain similar and the results from this simulation can also be used for flow modelling within the device.

Another major difference between the simulated particles and bacteria is the ability to anchor to surfaces. This unfortunately cannot be simulated and can only be inferred upon. Based on the flow simulations, it is confirmed that the flow will pull in the bacteria into the branch channels and subsequently the side channels. Upon this, the bacteria will stick unto the walls of the branches and floor of the side channels as the height of the branch microchannels here are approximately 1.5μm which is the width of a typical bacilli. This is atypical of bacteria adhesion. With the increased exposure to the surfaces, the bacteria adhesion induces a resistance against flow which thus acts as an inertial force that accordingly results in the trapping in the bottom of the side channels.

References

1. Tanaka, D.; Ishihara, J.; Takahashi, H.; Kobayashi, M.; Miyazaki, A.; Kajiya, S.; Fujita, R.; Maekawa, N.; Yamazaki, Y.; Takaya, A.; et al. High-Efficiency Single-Cell Containment Microdevices Based on Fluid Control. *Micromachines* **2023**, *14*, doi:10.3390/mi14051027.
2. Khaji, Z.; Tenje, M. Integrated cooling system for microfluidic PDMS devices used in biological microscopy studies. *Journal of Micromechanics and Microengineering* **2022**, *32*, doi:10.1088/1361-6439/ac7772.
3. Forsyth, B.; Torab, P.; Lee, J.H.; Malcom, T.; Wang, T.H.; Liao, J.C.; Yang, S.; Kvam, E.; Puleo, C.; Wong, P.K. A rapid single-cell antimicrobial susceptibility testing workflow for bloodstream infections. *Biosensors* **2021**, *11*, doi:10.3390/bios11080288.
4. Cama, J.; Voliotis, M.; Metz, J.; Smith, A.; Iannucci, J.; Keyser, U.F.; Tsaneva-Atanasova, K.; Pagliara, S. Single-cell microfluidics facilitates the rapid quantification of antibiotic accumulation in Gram-negative bacteria. *Lab on a Chip* **2020**, *20*, 2765-2775.
5. Long, Z.; Nugent, E.; Javer, A.; Cicuta, P.; Sclavi, B.; Cosentino Lagomarsino, M.; Dorfman, K.D. Microfluidic chemostat for measuring single cell dynamics in bacteria. *Lab on a Chip* **2013**, *13*, 947-954, doi:10.1039/c2lc41196b.

6. Wang, P.; Robert, L.; Pelletier, J.; Dang, W.L.; Taddei, F.; Wright, A.; Jun, S. Robust growth of Escherichia coli. *Current biology* **2010**, *20*, 1099-1103.
7. Asayesh, F.; Zarabadi, M.P.; Greener, J. A new look at bubbles during biofilm inoculation reveals pronounced effects on growth and patterning. *Biomicrofluidics* **2017**, *11*, doi:10.1063/1.5005932.
8. Zheng, H.; Yan, N.; Feng, W.; Liu, Y.; Luo, H.; Jing, G. Swimming of Buoyant Bacteria in Quiescent Medium and Shear Flows. *Langmuir* **2023**, *39*, 4224-4232, doi:10.1021/acs.langmuir.2c03088.
9. Spagnolie, S.E.; Moreno-Flores, G.R.; Bartolo, D.; Lauga, E. Geometric capture and escape of a microswimmer colliding with an obstacle. *Soft Matter* **2015**, *11*, 3396-3411, doi:10.1039/c4sm02785j.
10. Savorana, G.; Geisel, S.; Cen, T.; Ling, Y.; Stocker, R.; Rusconi, R.; Secchi, E. Transport of Pseudomonas aeruginosa in Polymer Solutions. *Frontiers in Physics* **2022**, *10*, doi:10.3389/fphy.2022.910882.
11. Chang, K.W.; Cheng, H.W.; Shiue, J.; Wang, J.K.; Wang, Y.L.; Huang, N.T. Antibiotic Susceptibility Test with Surface-Enhanced Raman Scattering in a Microfluidic System. *Analytical Chemistry* **2019**, *91*, 10988-10995, doi:10.1021/acs.analchem.9b01027.
12. Krafft, B.; Tycova, A.; Urban, R.D.; Dusny, C.; Belder, D. Microfluidic device for concentration and SERS-based detection of bacteria in drinking water. *Electrophoresis* **2021**, *42*, 86-94, doi:10.1002/elps.202000048.
13. Nam, Y.H.; Lee, S.K.; Kim, J.H.; Park, J.H. PDMS membrane filter with nano-slit array fabricated using three-dimensional silicon mold for the concentration of particles with bacterial size range. *Microelectronic Engineering* **2019**, *215*, doi:10.1016/j.mee.2019.111008.
14. Kim, M.C.; Klapperich, C. A new method for simulating the motion of individual ellipsoidal bacteria in microfluidic devices. *Lab on a Chip* **2010**, *10*, 2464-2471, doi:10.1039/c003627g.
15. Liao, C.C.; Chen, Y.Z.; Lin, S.J.; Cheng, H.W.; Wang, J.K.; Wang, Y.L.; Han, Y.Y.; Huang, N.T. A microfluidic microwell device operated by the automated microfluidic control system for surface-enhanced Raman scattering-based antimicrobial susceptibility testing. *Biosensors and Bioelectronics* **2021**, *191*, doi:10.1016/j.bios.2021.113483.
16. Tokárová, V.; Perumal, A.S.; Nayak, M.; Shum, H.; Kašpar, O.; Rajendran, K.; Mohammadi, M.; Tremblay, C.; Gaffney, E.A.; Martel, S.; et al. Patterns of bacterial motility in microfluidics-confining environments. *Proceedings of the National Academy of Sciences of the United States of America* **2021**, *118*, doi:10.1073/pnas.2013925118.
17. Vasdekis, A.E. Single microbe trap and release in sub-microfluidics. *RSC Advances* **2013**, *3*, 6343-6346, doi:10.1039/c3ra40369f.
18. Molaei, M.; Sheng, J. Succeed escape: Flow shear promotes tumbling of Escherichia colinear a solid surface. *Scientific Reports* **2016**, *6*, doi:10.1038/srep35290.
19. Ganzinger, K.A.; Merino-Salomón, A.; García-Soriano, D.A.; Butterfield, A.N.; Litschel, T.; Siedler, F.; Schwille, P. FtsZ Reorganization Facilitates Deformation of Giant Vesicles in Microfluidic Traps**. *Angewandte Chemie - International Edition* **2020**, *59*, 21372-21376, doi:10.1002/anie.202001928.
20. Gruenberger, A.; Probst, C.; Heyer, A.; Wiechert, W.; Frunzke, J.; Kohlheyer, D. Microfluidic picoliter bioreactor for microbial single-cell analysis: fabrication, system setup, and operation. *Journal of visualized experiments : JoVE* **2013**, 50560, doi:10.3791/50560.
21. Galon, C.M.V.; Madriaga, M.R.A.; Margaja, I.B. Trapping bacteria and fungi using microfluidic design. *International Journal of Advanced and Applied Sciences* **2022**, *9*, 110-116, doi:10.21833/ijaas.2022.01.013.
22. Probst, C.; Grünberger, A.; Wiechert, W.; Kohlheyer, D. Polydimethylsiloxane (PDMS) sub-micron traps for single-cell analysis of bacteria. *Micromachines* **2013**, *4*, 357-369, doi:10.3390/mi4040357.
23. Chatzimichail, S.; Turner, P.; Feehily, C.; Farrar, A.; Crook, D.; Andersson, M.; Oakley, S.; Barrett, L.; El Sayyed, H.; Kyropoulos, J.; et al. Rapid identification of bacterial isolates using

microfluidic adaptive channels and multiplexed fluorescence microscopy. *Lab on a Chip* **2024**, *24*, 4843-4858, doi:10.1039/d4lc00325j.
24. Zhang, B.; Wu, W.; Zhao, Q.; Yan, S. Geometric optimization of double layered microchannel with grooves array for enabling nanoparticle manipulation. *Physics of Fluids* **2023**, *35*, doi:10.1063/5.0152929.
25. Zhang, B.; Zou, S.; Wu, W.; Yang, F.; Mehmood, K.; Wan, W.; Zhao, Q. Mechanism and Effects of Cellular Creep in a Microfluidic Filter. *Journal of Physical Chemistry Letters* **2022**, *13*, 8641-8647, doi:10.1021/acs.jpclett.2c02137.
26. Wen, K.; Gorbushina, A.A.; Schwibbert, K.; Bell, J. Microfluidic Platform with Precisely Controlled Hydrodynamic Parameters and Integrated Features for Generation of Microvortices to Accurately Form and Monitor Biofilms in Flow. *ACS Biomaterials Science and Engineering* **2024**, *10*, 4626-4634, doi:10.1021/acsbiomaterials.4c00101.
27. Tanasijević, I.; Lauga, E. Microswimmers in vortices: dynamics and trapping. *Soft Matter* **2022**, *18*, 8931-8944, doi:10.1039/d2sm00907b.
